# Secondary Hemophagocytic Syndrome: The Importance of Clinical Suspicion

**DOI:** 10.1155/2014/958425

**Published:** 2014-05-19

**Authors:** Cristina Oliveira, Sérgio Chacim, Isabel Ferreira, Nelson Domingues, José Mário Mariz

**Affiliations:** ^1^Oncology Department, Portuguese Institute of Oncology of Porto, Rua Dr. António Bernardino de Almeida, 4200-072 Porto, Portugal; ^2^Hematology Department, Portuguese Institute of Oncology of Porto, Rua Dr. António Bernardino de Almeida, 4200-072 Porto, Portugal

## Abstract

Hemophagocytic syndrome is a rare and potentially fatal disorder characterized by pathological immune activation associated with a primary familial disorder, genetic mutations, or occurring as a sporadic condition. The latter can be secondary to infections, malignancies, or autoimmune diseases. Clinically, patients present signs of severe inflammation, with unremitting fever, cytopenias, spleen enlargement, phagocytosis of bone marrow elements, hypertriglyceridemia, and hypofibrinogenemia. Increased suspicion is determinant to timely initiate treatment in an attempt to alter the natural history. The authors present three clinical cases of this syndrome, with a brief review of the diagnostic criteria and treatment.

## 1. Introduction

Hemophagocytic syndrome (HPS) is a potentially fatal hyperinflammatory disease induced by a pathologic immune activation, with proliferation of well-differentiated macrophages/histiocytes and increased phagocytic activity [1]. This entity was first described in 1939 and in 1952 was identified as an immune hereditary disorder, familial hemophagocytic lymphohistiocytosis (FHL) [1, 2].

HPS affects approximately 1.2 million people per year. However, incidence can be underestimated since diagnosis is often missed [3].

FHL is associated with mutations in genes with an autosomal recessive trait. When HPS is the only manifestation of the disease, in FHL 2–5 the related mutations are* PRF1*,* UNC13D*,* STX11*, and* STXBP2*, respectively. All these genes encode proteins involved in lymphocyte cytotoxicity [4]. Some hereditary diseases associated with partial albinism, such as Griscelli syndrome type 2 (mutations in* RAB27A *gene), Chediak-Higashi (mutations in* LYST *gene), and Hermansky-Pudlak 2 (mutations* AP3B1 *gene), also predispose to HPS [3, 4]. Secondary forms are associated with infections (with EBV being the most common), collagen vascular diseases, and malignancies, particularly lymphomas and leukemias [2, 3, 5, 6]. The most commonly reported hematologic malignancies are NK or T lymphomas or leukemias. Solid tumors are a less frequent cause [2].

The pathogenesis of HPS remains poorly understood; however, uncontrolled macrophage and T-helper 1 (Th-1) lymphocyte activation seems to be crucial. Overproduction of cytokines involved in Th-1 lymphocyte and macrophage activation, such as interferon-*α*, soluble interleukin-2-receptor (sIL-2R), tumor necrosis factor, IL-1, or IL-6, has been consistently reported. An alternative hypothesis involves failure to remove antigen, which results in ongoing stimulation of the immune system [3, 6].

The clinical features of HPS include unremitting high fever, hepatosplenomegaly, cytopenias, high fasting triglycerides, and ferritin levels. The hallmarks of the disease are usually found in the bone marrow with the presence of numerous well-differentiated macrophages phagocytosing hematopoietic cells [6]. However, diagnosing HPS is difficult and it is a critical step because of its rare occurrence, variable presentation, and nonspecific findings, easily assigned to more common entities [1].

## 2. Case 1

**Figure 1:**
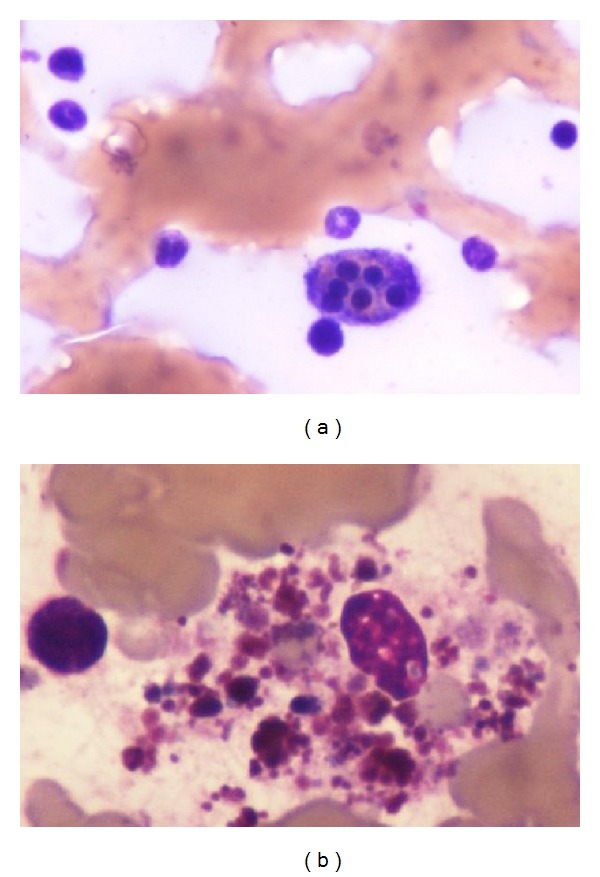
(a) Bone marrow aspiration of patient 1 and (b) bone marrow aspiration of patient 2, both showing macrophages phagocyting blood elements, hallmark of HPS.

A 75-year-old man, treated for arterial hypertension, was diagnosed with stage I colon cancer. He was submitted to colon resection and one week later developed an infectious peritonitis, that was controlled with systemic broad spectrum antibiotics. One month after surgery, he presents to his general practitioner (GP) with daily fever (>38°C), weight loss, and progressive asthenia. Physical examination was not remarkable. The GP suspected infection and prescribed oral antibiotics. Blood tests showed pancytopenia (Table 2) with negative serology for HIV, B and C hepatitis, EBV, CMV,* Rickettsia conorii, Leptospira, Borrelia burgdorferi,* and* Salmonella typhi.* Active tuberculosis infection was also excluded. The condition extended for 3 months, with no response to treatment and clinical deterioration with unremitting fever. The patient was admitted to hospital where he began broad spectrum antibiotics (piperacillin-tazobactam 4,5 g, t.i.d.) and prednisolone 40 mg/m^2^. Repeated serologies and bacteriologic cultures were inconclusive. A thoracoabdominal CT scan showed a soft spleen enlargement (14,5 cm × 6 cm). The main clinical suspicion was an occult infection. Blood tests showed persistent and worsening pancytopenia, with hepatic cytolysis and cholestasis, elevated ferritin, and triglycerides (Table 2). A bone marrow biopsy was performed presenting signs of phagocytosis of blood elements. The diagnosis of HPS was then made (Figure 1). Despite the diagnosis, his clinical status worsened and rapidly evolved to multiorgan failure (MOF) with hepatic, respiratory, and cardiac dysfunction. He died 10 days after admission.

## 3. Case 2

A 62-year-old woman with a previous history of bone tuberculosis in childhood presented with lymph node tuberculosis reactivation. She initiated therapy (isoniazid, pyrazinamide, rifampicin, and ethambutol), but after ten months treatment was interrupted due to pancytopenia. Drug toxicity was suspected, but pancytopenia persisted after stopping the treatment. A first bone marrow biopsy was inconclusive. The patient's hematologic cell counts continue to drop (Table 2) and a new medullar evaluation was compatible with a myelodysplastic syndrome with complex karyotype (chromosomes 5 and 7 deletions). Treatment with azacitidine was started and soon interrupted due to the presence of fever with absent signs of infection. The patient was admitted to hospital. The fever was refractory to antibiotics (imipenem, vancomycin) and fluconazole. The bacteriologic studies of urine, respiratory secretions, and blood cultures were negative. A high ferritin value of 19000 *μ*g/L made the suspicion of HPS. Further tests revealed elevated *α*-chain IL-2 soluble receptor and low fibrinogen levels (Table 2). A new bone marrow biopsy revealed signs of hemophagocytosis (Figure 1). At this point, the diagnosis of HPS was made. Even though the patient did not present neurologic symptoms, a lumbar puncture was performed revealing high protein levels, indicating probable central nervous system (CNS) involvement. The patient began treatment with etoposide and dexamethasone and intrathecal methotrexate according to protocol HLH-94 [3] with clinical improvement, resolution of fever, and continuous recovery of blood count (Table 2). She completed 8 weeks of therapy. Despite an initial clinical improvement, she was readmitted to hospital and died with septic shock due to a severe respiratory infection 2 months after completion of initial therapy.

## 4. Case 3

A 66-year-old man with no relevant medical history began fever, night sweats, and odynophagia, associated with submandibular lymph node growth. Blood tests were remarkable for pancytopenia with elevated levels of ferritin, triglycerides, and liver enzymes with signs of liver dysfunction and coagulopathy (Table 2). A thoracoabdominal CT scan revealed multiple enlarged lymph nodes, hepatic lesions, bilateral pleural effusions, and ascites. Biopsy of the submandibular node was compatible with non-Hodgkin lymphoma of peripheral T cells (CD3+, CD20−, CD5−, CD10−, CD30−, and ALK−). Bone marrow biopsy showed hemophagocytosis and no involvement by lymphoma. The patient initiated treatment with methylprednisolone and etoposide with no obvious response and progression to multiorgan dysfunction and death in a few days.

## 5. Discussion

Hemophagocytic syndrome is a rare hyperinflammatory disease, with a dismal prognosis if not promptly treated [6]. High clinical suspicion and early diagnosis are of outmost importance and a clinical challenge. The diagnosis is suspected from clinical and laboratory criteria, proposed by the Histiocyte Society, and is made in the presence of at least 5 of 8 criteria (Table 1) or established in the presence of specific genetic mutations [1, 2]. The utility of these criteria is questionable because they lack specificity. However, some authors argue that despite the lack of specificity of individual criteria the ensemble that reflects disease severity is the crucial point [3]. The literature suggests that ferritin levels >10 000 *μ*g/L are highly sensitive and specific for the diagnosis of HPS, and levels >30 000 *μ*g/L can be 100% specific in the absence of iron metabolism disturbances. However, not all patients have hemophagocytosis at disease onset and the diagnosis should not be delayed because of this [1–3]. Fever and spleen enlargement are present in about 75% of patients at time of diagnosis and bicytopenia, hypertriglyceridemia, and ferritin >500 *μ*g/L are present in half of the patients. About one-third can present with CNS involvement, and, in the presence of any neurologic symptoms, it should promptly be excluded [2]. In summary, unexplained fever, liver failure with concurrent cytopenias, and elevated inflammatory indices should alert the clinician for the diagnosis of HPS [1].

All three cases presented are secondary forms of HPS. In case 1, HPS seems to be secondary to abdominal peritonitis, which likely triggered immune activation. Colon cancer was a less probable cause since solid malignancies rarely cause HPS [2]. In addition, in this case, colon cancer was in a very early stage, and the disease was promptly controlled by surgery. The differential diagnosis was mostly with infectious disease, because clinical findings and symptoms could mimic a septic process. Despite all the investigations, no infection was identified. The unusual high levels of ferritin make HPS a possible cause for the clinical condition described. In this case, 6 of the 8 HPS criteria were present. Levels of fibrinogen and *α*-chain of soluble IL-2 receptor were not measured, due to the rapid evolution and clinical deterioration.

In case 2, myelodysplastic syndrome assumes a relevant role as a possible trigger of HPS. Reactivation of tuberculosis was also considered since infections are known triggers of HPS. Pancytopenia was installed during ongoing tuberculosis treatment, in a phase where infection was not yet controlled.

However, a diagnosis of myelodysplastic syndrome was also made, and the development of prolonged fever not responding to antibiotics and no infectious focus, together with the elevated ferritin, raise the possibility of the presence of another entity beside myelodysplastic syndrome, HPS. When this diagnosis was suspected, the investigation was completed with the measurement of the *α*-chain IL-2 soluble receptor. In this case, 6 of 8 diagnostic criteria were also present.

In case 3, T cell lymphoma is the most likely culprit and HPS was a simultaneous diagnosis. The patient also presented with high levels of ferritin. However, in the initial phase, lymphoma was thought to be responsible for the patient's condition and the diagnosis of HPS came later with medullar evaluation, needed for staging T cell lymphoma. The rapid deterioration and dismal outcome were more likely attributable to HPS than to the lymphoma itself and T cell lymphomas are one of the most common hematologic causes of HPS. In this case, 5 of 8 criteria of HPS were present. When diagnosis was made, the patient initiated etoposide and corticosteroids treatment, but, due to the fast progression to multiorgan failure, probably because the immune cascade could not be controlled anymore, the patient died in a few days.

All of our cases had at least 5 of the 8 criteria of HPS. Although the diagnosis was made in the three cases, in two of them, it was not possible to initiate treatment on time, maybe not only because of the advanced and accelerated phase of the inflammatory cascade, impossible to revert, but also due to a delayed diagnosis, especially in cases 1 and 3, and the rapid clinical deterioration. In all the cases, and as described in the literature, the unusual elevated levels of ferritin were the discriminatory sign and the crucial point to suspect HPS, because the other symptoms and criteria lack sensitivity and specificity to HPS and can be present in many other clinical entities. Levels of ferritin as high as those verified here are present in patients with iron metabolism disease, which was not fit or plausible in any of those cases. However, the moment of confirmation of the diagnosis in all the cases was with the evidence of phagocytosis in bone marrow.

The three cases described here represent the dismal prognosis of HPS with fatal outcome in a short period of time, reflecting fast disease progression when diagnosis is not timely suspected and effective treatment promptly started. Despite the fact that prognosis of HPS improved in the last years, it still remains very poor with 50% mortality and with a two-month survival if left untreated [6]. Because of that, it is essential not to delay treatment while waiting for the results of diagnostic tests. Since the introduction of the first international protocol, by the Histiocyte Society in 1994 (HLH-94), prognosis has improved, with an overall survival of 55%, with 3.1 years of followup. This protocol consists in a combination of dexamethasone, etoposide, and intrathecal methotrexate for 8 weeks. Only when etoposide, a proapoptotic chemotherapy drug, was added, were sustained remissions described [3]. Attending to the response obtained, patients can continue with the same treatment or can be submitted to allogeneic stem cell transplantation (ASCT). Generally ASCT is recommended on documented FHL, recurrent or progressive disease despite intensive therapy, and CNS involvement [1]. It is important to note that, in cases of secondary HPS, the treatment of the underlying cause is critical to control disease progression. However, with the exception of autoimmune disease and malignancy, initial therapy for patients with suspected familial or reactive HPS should be the same [1].

In conclusion, the authors highlight the importance of high clinical suspicion of this syndrome, despite its rarity and complexity. Levels of ferritin are simple and inexpensive to measure and can greatly increase the suspicion of HPS, with unusual high values.

A high index of suspicion in selected patients remains the most powerful tool for the diagnosis and timely treatment of the disease. Further research on the pathophysiology of HPS is needed to support the development of better treatments that can improve patient outcome [1].

## Figures and Tables

**Table 1:** Diagnosis criteria of HPS according to HLH-2004.

Diagnosis of HPS	
(A) Molecular diagnosis consistent with HPS: *PRF1*, *UNC13D*, *Munc18-2*, *Rab27a*, *STX11*, *SH2D1A*, or *BIRC4 *	
Or	
(B) 5 of 8 diagnostic criteria below:	
(1) fever ≥ 38,5°C	
(2) spleen enlargement	
(3) cytopenias affecting at least 2 blood lineages in peripheral blood:	
(a) hemoglobin < 9,0 g/dL	
(b) platelets < 100 × 10^9^	
(c) absolute neutrophil count < 1,0 × 10^9^	
(4) elevated triglycerides (fasting > 265 mg/dL) and/or hypofibrinogenemia (<150 mg/dL)	
(5) hemophagocytosis in bone marrow, spleen, lymph nodes, or liver	
(6) absent or low NK-cell activity	
(7) ferritin > 500 *μ*g/L (>10 000 *μ*g/L highly suspicious of HPS)	
(8) elevated sCD25 (*α*-chain of soluble IL-2 receptor) > 2400 U/mL	

Adapted from Jordan et al. [1].

**Table 2:** Description of patients according to probable trigger of HPS, diagnostic criteria of HPS, treatment, and evolution.

	Patient 1	Patient 2	Patient 3
Probable trigger of HPS	Abdominal peritonitis	Active tuberculosis or myelodysplastic syndrome	T cell lymphoma

Blood analyses evolution	Hemoglobin 8,8 g/dL→7 g/dL Leucocytes > 3 × 10^9^/L→1,23 × 10^9^/L Platelet 140 × 10^9^/L→29 × 10^9^/L Ferritin 1085 *μ*g/L→21091 *μ*g/L Fasting triglycerides 329 mg/dL AST 84 U/L Alkaline phosphatase 192 U/L C-reactive protein 210 mg/L	Hemoglobin 5,8 g/dL→5 g/dL Leucocyte 3 × 10^9^/L→0,9 × 10^9^/L Platelet 80 × 10^9^/L→3 × 10^9^/L Ferritin 19 000 *μ*g/L Fibrinogen 177 mg/dL (NR 200–400 mg/dL) *α*-Chain of sIL-2—2377 U/mL (NR 158–623 U/mL) **After initial treatment** Hemoglobin 9,5 g/dL Leucocytes 3,35 × 10^9^/L Platelet 75 × 10^9^/L	Hemoglobin 11,6 g/dL→7,8 g/dL Leucocyte 2,5 × 10^9^/L→0,5 × 10^9^/L Platelet 188 × 10^9^/L→18 × 10^9^/L Ferritin 11973 *μ*g/L Fasting triglycerides 345 mg/dL Total bilirubin 18,12 mg/dL Direct bilirubin 14,2 mg/dL ALT 90 U/L Fibrinogen < 20 mg/dL

Unremitting fever	Yes	Yes	Yes

Spleen enlargement	Yes	No	No

Medullar phagocytosis	Yes	Yes	Yes

Number of HPS criteria	6 in 8	6 in 8	5 in 8

Neurologic symptoms	No	No	No

Involvement of CNS	Unknown	Yes	Unknown

Treatment realized to HPS	Prednisolone 40 mg/m^2^	(i) Dexamethasone for 8 weeks with tapering dose from 10 mg/m^2^ to 1,25 mg/m^2^ dose (ii) Etoposide 150 mg/m^2^ for 8 weeks (iii) Intrathecal methotrexate (12 mg) in weeks 2, 3, 4, and 5	(i) Methylprednisolone (ii) Etoposide 150 mg/m^2^

Evolution	Dead in 3 days after diagnosis (10 days after admission)	Dead in 4 months after diagnosis	Dead in 4 days after diagnosis (22 days after admission)
